# Parvalbumin increases in the medial and lateral geniculate nuclei of aged rhesus macaques

**DOI:** 10.3389/fnagi.2013.00069

**Published:** 2013-11-08

**Authors:** Daniel T. Gray, Megan L. Rudolph, James R. Engle, Gregg H. Recanzone

**Affiliations:** ^1^Center for Neuroscience, University of California at DavisDavis, CA, USA; ^2^Evelyn F. McKnight Brain Institute, University of ArizonaTucson, AZ, USA; ^3^Department of Neurobiology, Physiology and Behavior, University of California at DavisDavis, CA, USA

**Keywords:** parvalbumin, thalamus, medial geniculate, lateral geniculate, monkey, geriatric

## Abstract

Subcortical auditory structures in the macaque auditory system increase their densities of neurons expressing the calcium binding protein parvalbumin (PV) with age. However, it is unknown whether these increases occur in the thalamic division of the auditory system, the medial geniculate nucleus (MGN). Furthermore, it is also unclear whether these age-related changes are specific to the macaque auditory system or are generalized to other sensory systems. To address these questions, the PV immunoreactivity of the medial and lateral geniculate nuclei (LGN) from seven rhesus macaques ranging in age from 15 to 35 was assessed. Densities of PV expressing neurons in the three subdivisions of the MGN and the six layers of the LGN were calculated separately using unbiased stereological sampling techniques. We found that the ventral and magnocellular subdivisions of the MGN and all six layers of the LGN increased their expressions of PV with age, although increases in the MGN were greater in magnitude than in the LGN. Together, these results suggest that the MGN shows age-related increases in PV expression as is seen throughout the macaque ascending auditory system, and that the analogous region of the visual system shows smaller increases. We conclude that, while there are some similarities between sensory systems, the age-related neurochemical changes seen throughout the macaque auditory system cannot be fully generalized to other sensory systems.

## INTRODUCTION

Parvalbumin (PV) immunoreactivity is a well-established neurochemical marker of subsets of neurons in several sensory systems of the mammalian brain (Demeulemeester et al., 1989; Celio, 1990; Hof et al., 1999; Jones, 2003; Dávid et al., 2007). PV and other calcium binding proteins have calcium-buffering capacities, and several types of cortical interneurons are classified by their chemical signatures to these molecules (Carder et al., 1996). Intracellular calcium affects numerous calcium dependent biochemical pathways, most of which alter the physiological environment of the cell. These calcium-buffering proteins therefore indirectly regulate intracellular processes through their control of free calcium levels (Yáñez et al., 2012). Furthermore, neurotransmitter release is calcium dependent (Tang et al., 2011), and these molecules likely affect this process to some degree as well. A coarse sense of changes in the intracellular environment and signaling capabilities of neurons can be attained by using histological markers for calcium binding proteins as chemical probes, and noting changes in their expression under different conditions, in this case natural aging.

The relative density of PV+ neurons can be altered as a function of aging in some sensory systems, and also in animals given peripheral insults mimicking the effects of aging. The majority of these observations come from rodent models (O’Neill et al., 1997; Zettel et al., 1997; Idrizbegovic et al., 2003, 2004, 2006; Ouda et al., 2008; Hatano et al., 2009; Ouda et al., 2012), but recently the rhesus macaque has emerged as another model to study these age-related changes. The macaque auditory and visual systems contain PV+ neurons at subcortical and cortical levels, and both systems show changes in PV immunoreactivity with natural aging or conditions associated with aging (Blümcke et al., 1991; Glezer et al., 1998). For example, within the auditory system, presbycusis (age-related hearing loss) in the macaque monkey is correlated with increases in the numbers of PV labeled neurons in the superior olivary complex (SOC) and the inferior colliculus (IC; Gray et al., 2013a; unpublished observations), but not in the cochlear nucleus (CN; Gray et al., 2013b), and it remains is unknown whether these changes occur in the macaque MGN or not. In the visual system, binocular and/or monocular enucleation of adult rhesus monkeys results with decreases in PV immunoreactivity in the neuropil of the LGN and primary visual cortex (Blümcke et al., 1994), and similar decreases have been noted in glaucoma patients (Yücel et al., 2000). The functional consequences of these changes remain unclear, but they are thought to play a role in compensating for the deficits in natural processing that are associated with age.

The medial (MGN) and lateral (LGN) geniculate nuclei are the principle thalamic divisions of the auditory and visual systems, respectively. The extraction of basic spatial and spectral information occurs upstream from the thalamus in both systems, therefore sensory information arriving to both of these nuclei contains similar information (i.e., what the stimulus is and where the stimulus came from). For example, the SOC in the auditory brainstem contains neurons sensitive to spatial cues, and spatial sensitivity to sounds in azimuth and elevation are found in the IC in the midbrain (Moore, 1991). In the visual system, the majority of this processing occurs in the retina (Kuffler, 1953; Rodieck and Stone, 1965; Lee, 1996). Similarly, most spectral encoding occurs early in the processing stream of both systems at the level of the inner hair cells along the basilar membrane of the cochlea and the photoreceptors of the retina (Robles and Ruggero, 2001; Conway, 2009). While both the visual and auditory cortices continue to represent and further process spatial and spectral information (Shapley and Hawken, 2002; Conway, 2009; Recanzone, 2011; Adesnik et al., 2012), by the thalamic division of both sensory systems the foundations of this information has been extracted. Therefore, the MGN and LGN can be viewed as anatomically and functionally analogous regions of the auditory and visual systems, making the geniculate a good comparison site between these two sensory pathways.

Changes in PV immunoreactivity have been studied in the auditory system, but not in the visual system of naturally aging macaque monkeys. Comparing analogous regions of the visual and auditory systems could be useful in determining if the neurochemical changes that are employed by the auditory system as a consequence of aging are used by multiple sensory systems, or if these changes are specific to the auditory system. Therefore, in the present study we quantify the age-related changes in PV immunoreactivity within the primary thalamic divisions of the auditory and visual systems, the MGN and LGN respectively. We partitioned the MGN into its three principle subdivisions, the ventral MGN (vMGN), dorsal MGN (dMGN), and the magnocellular MGN (mMGN), and quantified the density of PV+ cells separately for each subdivision. Similarly, the density of PV+ neurons of the two magnocellular and four parvocellular layers of the LGN were quantified separately to compare the ipsilateral and contralateral representations.

## METHODS

The MGN and LGN from seven rhesus macaques ranging from 15 to 35 years of age (roughly equivalent to 37 to 107 human years of age; see Davis and Leathers, 1985) were quantified to determine the densities of PV expressing neurons using unbiased stereological sampling techniques. **Table 1** describes the demographic information of all animals used in this analysis. No animal had a history of loud noise exposure, ear trauma, or ototoxic drug treatment. No monkeys had obvious visual impairments or ocular abnormalities, although this was not rigorously tested. All monkeys were maintained on ad libitum food and water with the exception of one (25 year old) who was briefly water regulated during training on auditory discrimination tasks. All procedures adhered to the National Institute of Health’s guidelines, and were approved by the UC Davis Institutional Animal Care and Use Committee.

**Table 1:** Demographics and information on the monkeys used.

Age (months)	Human age (years)	Gender	Thickness (μm)	Auditory training	Group
417	37	Male	25	No	Middle-aged
243	61	Female	50	Yes	Middle-aged
245	61	Female	40	No	Middle-aged
267	67	Female	50	No	Aged
276	69	Female	50	No	Aged
306	76.5	Male	50	Yes	Aged
427	107	Female	25	No	Aged

### HISTOLOGICAL PROCESSING AND ANTIBODY CHARACTERIZATION

Histological processing for PV immunohistochemistry has been previously described in detail (Gray et al., 2013a). Briefly, animals were euthanized with an overdose of sodium pentobarbital (60 mg/kg, i.v.), and transcardially perfused with a solution of 4% paraformaldehyde, 0.1% glutaraldehyde (for cross-linking of macromolecules), and saline in 0.1% phosphate buffer (pH 7.4; fix 1), and a mixture of 4% paraformaldehyde and 10% sucrose to initiate cryoprotection (fix 2). Following perfusion, the brains were extracted and placed in a solution of 4% paraformaldehyde and 30% sucrose for complete cryoprotection. All seven brains were cut transversely at thicknesses ranging from 25–50 μm. The tissue was stored in 0.1 phosphate buffer until tissue processing (Hackett et al., 2001; de la Mothe et al., 2006a,b; Padberg et al., 2009), which occurred within 2 months of euthanasia. Sections were alternately stained for PV immunohistochemistry and Nissl.

Parvalbumin immunohistochemistry followed a modified protocol of the ABC method (ABC kit, Vector Labs), and visualized with the 3, 3′-diaminobenzidine (DAB) and hydrogen peroxide reaction. The sections were blocked overnight (12 h) in 3% normal horse serum and 0.25% Triton X, followed by a 4 h incubation in the primary PV antibody (anti-PV in mouse, 1:4000; Sigma-Aldrich). Following several washes in 0.1% phosphate buffer the sections were incubated for 1 h in the secondary antibody (Biotinylated Anti-Mouse IgG, 1:250; Vector Labs). After several more rinses in phosphate buffer, the sections were placed in a peroxidase substrate solution for the DAB reaction until the desired intensity, yielding good label with minimal background staining, was achieved (5–8 min). The Sigma immunoType^™^ Kit (Product Code ISO-1), and a double diffusion immunoassay using Mouse Monoclonal Antibody Isotyping Reagents (Product Code ISO-2) confirmed isotype specificity of the anti-PV antibody as frog leg PV. Exclusion of the primary antibody yielded only weak background staining with no punctate signal in any animal, indicating that all positive signals resulted from the PV antibody, and not extraneous label. The information for all antibodies and chemicals used in this procedure is summarized in **Table 2**.

**Table 2:** Information on the antibodies and chemicals used for immunohistochemical and histochemical reactions.

	Antibody/chemical	Immunogen structure	Manufacturer/Log #/other informatiion
Parvalbumin immunohistochemistry	Monoclonal anti-parvalbumin clone PARV-19	PARV-19 hybridoma	Sigma-Aldrich, P-3088; monoclonal; raised in mouse
Parvalbumin immunohistochemistry	Biotinylated anti-Mouse IgG	Mouse IgG	Vector Labs, BA-2000; made in horse
Parvalbumin immunohistochemistry	Normal horse serum	N/A	Vector Labs, S-2000
Parvalbumin immunohistochemistry	SG-substrate kit	N/A	Vector Labs SK-4700

### DATA ANALYSIS

Standard light microscopy and unbiased stereological sampling techniques were used to estimate the PV+ cell density of the MGN and LGN. These nuclei were sampled by two observers blind to the identity and age of the animals. A 0.5 mm^2^ grid was digitally overlaid on a 40× image of the tissue. This high magnification was chosen to ensure that the counting grid fell entirely within the boundaries of the layers of the LGN to avoid biases arising from differences in counting area. Differences in tissue thickness can bias cell counts such that thinner sections yield overestimations (Abercrombie and Johnson, 1946). Therefore we applied an optical fractionation approach in our density estimations. This procedure uses an optical disector with optimal guard spaces on either side of the z-plane to scan the depth of the tissue. Thus, fragmented cells at the edges of the tissue play a negligible part in the density calculation (West et al., 1991; Mounton, 2002). Estimated PV+ neurons (*E*_n_) were calculated as follows:

E_n_=*N*_v_ × *V*_ref_

Where,

*N*_v_=Σ*Q*/(*n*×*V*_dis_)

With *N*_v_ representing the points counted within the grid, *V*_ref_ is the volume calculated by the Cavalieri principle, *Q* is the cells counted, *n* is the number of disectors, and *V*_dis_ represents the volume of each disector. Since *N*_v_ and *V*_ref_ refer to a common volume, these calculations control for volume changes in the cell counts.

To ensure constancy in the counting criteria, the two observers counted non-geniculate sections until they reached a 95% agreement for 20 consecutive sections. A paired *t*-test of the estimates used for analysis revealed that the two observers did not differ (*p* > 0.4). Positively stained soma were defined as possessing a punctate cellular morphology containing positive label, and neurons were counted only if they fell entirely within the grid or if they touched two sides (top and right) of the grid. Several control analyses in previous studies using this same tissue and staining protocol ensured the observer’s ability to reliably distinguish labeled neurons from background staining of the neuropil (Gray et al., 2013a). The density of the sample was calculated by dividing the estimated cell count by the estimated volume (*E*_n_/*V*_ref_).

Statistical analysis was accomplished in three steps. First, the animals were grouped into middle-aged and aged groups using 21.66 years (corresponding to 65 human years; Davis and Leathers, 1985) as the cutoff. While each animal provided multiple measures of cell densities for each nucleus subdivision, the dataset was limited to three (middle-aged) and four (aged) animals. We therefore used relatively strict criteria to assess statistical significance in all of our analyses, and all *r*-values and *p*-values > 0.01 are reported as calculated. First, comparisons between groups were made using an unpaired *t*-test, with an alpha level of 0.01. Second, we used a linear regression model to understand the relationship of these densities as a function of age. In these calculations, the Pearson product moment correlation was found along with the *p*-value. We considered statistically significant trends to be those with *r*-values greater than 0.7 coupled with an alpha level of 0.01 or smaller. Finally, all age-density relationships underwent a Monte Carlo analysis. This analysis first calculates the *r*-values of the existing relationship, and then randomly re-assigns the age and density values before calculating the new *r*-value of the random relationship. This re-assignment is done 1000 times, and the *p*-value is the percentage of random relationships that give an *r*-value greater than or equal to the observed *r*-value. Therefore the Monte Carlo provides the probability that the observed relationship is due to chance. A *p*-value of 0.05 (i.e., a regression coefficient was greater than 950/1000 coefficients generated by chance) was used as the significance cutoff in this procedure. Relationships were only considered significant if they passed our criteria for both the regression analysis and the Monte Carlo analysis.

## RESULTS

**FIGURE 1:**
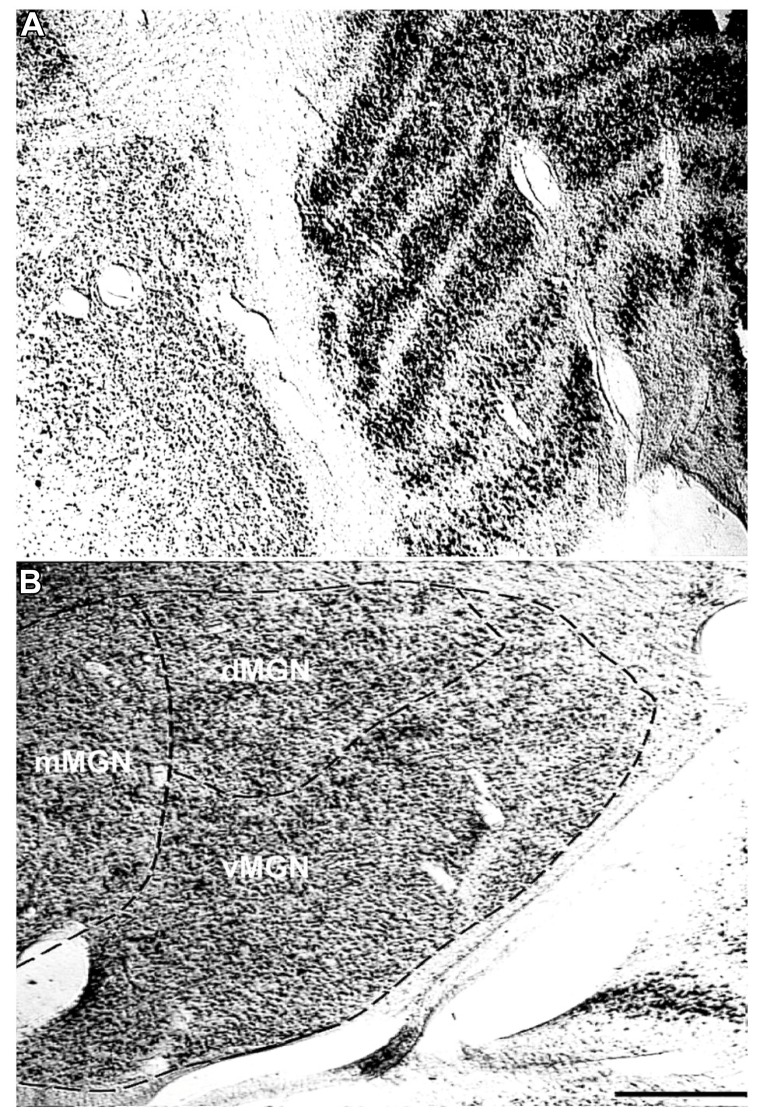
**Representative Nissl stains of the sensory thalamus.** **(A)** Transverse Nissl stained section showing the anatomical proximity of the medial and lateral geniculate nuclei. **(B)** The three subdivisions of the MGN, the ventral (vMGN), dorsal (dMGN), and magnocellular (mMGN) MGN, defined according to previous conventions (Jones, 2003). All MGN sections were partitioned in this manner for stereological quantifications. The left sides of these images correspond to the medial aspect and the top to the dorsal aspect of the sections. Scale bar = 500 μm.

To understand the age-related changes in PV expression within the MGN and LGN, serial sections from seven rhesus macaques ranging in age from 15 to 35 years were prepared (**Table 1**). The MGN and LGN both had positively stained neurons in all animals, and showed relatively little background staining of the neuropil. **Figure 1A** shows a Nissl stained section of both geniculate nuclei, and demonstrates the anatomical proximity of these two nuclei in the thalamus. The three principle subdivisions of the MGN, the vMGN, dMGN, and mMGN, were defined according to conventions of Jones (2003). The borders of these three subdivisions were not as readily apparent as the six layers of the LGN, making the delineation of these regions difficult. To ensure that all counts came entirely from within each subdivision we segregated every section analogously based on the conventions mentioned above (**Figure 1B**), and placed our counting grids away from these borders into regions that were unambiguously defined within a particular subdivision. The dMGN showed few neurons with PV staining, although those that did were clearly stained, an observation previously noted (Jones, 2003). The densities of PV+ stained cells, as measured by the number of positively stained neurons within a given volume of tissue (see Methods), were much greater in the other subdivisions of the MGN as well as in all six layers of the LGN.

**FIGURE 2:**
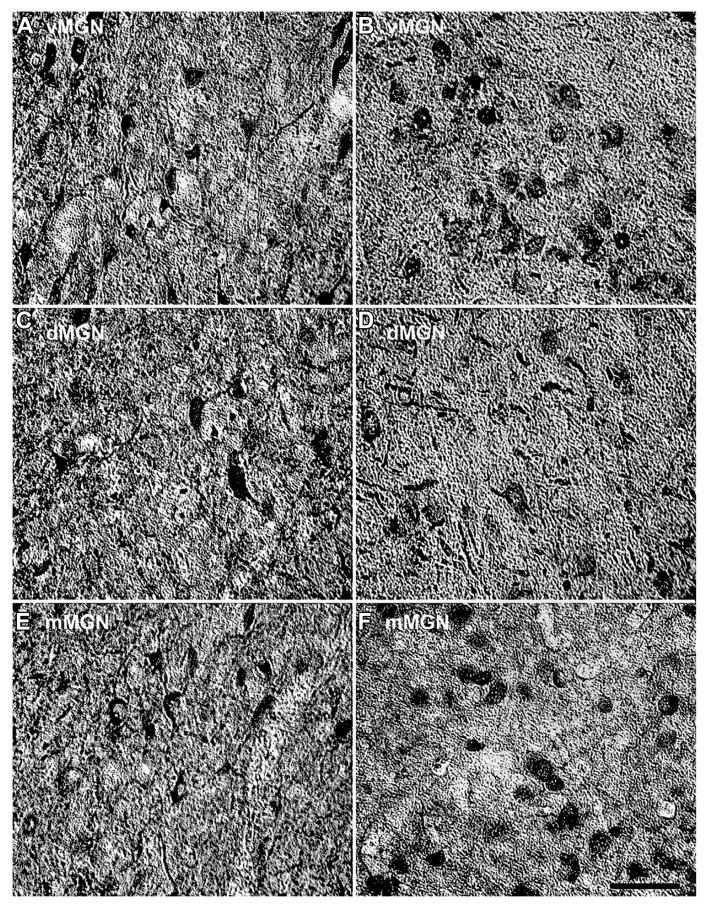
**Comparison of parvalbumin positive cell density of the MGN between the 15- and 35-year-old animals.** Comparison of micrographs from a **(A)** 15 year old vMGN and **(B)** 35 year old vMGN reveals apparent age-related increases in the number of stained cells. However, when comparing a **(C)** 15 year old dMGN with a **(D)** 35 year old dMGN, no apparent age-related changes in the number of stained neurons are seen. Similar to the vMGN, the mMGN showed age-related increases between the **(E)** 15 year old or and **(F)** 35 years old animals. The left side of this image corresponds to the medial aspect and the top to the dorsal aspect of the sections. Scale bar = 150 μm.

**FIGURE 3:**
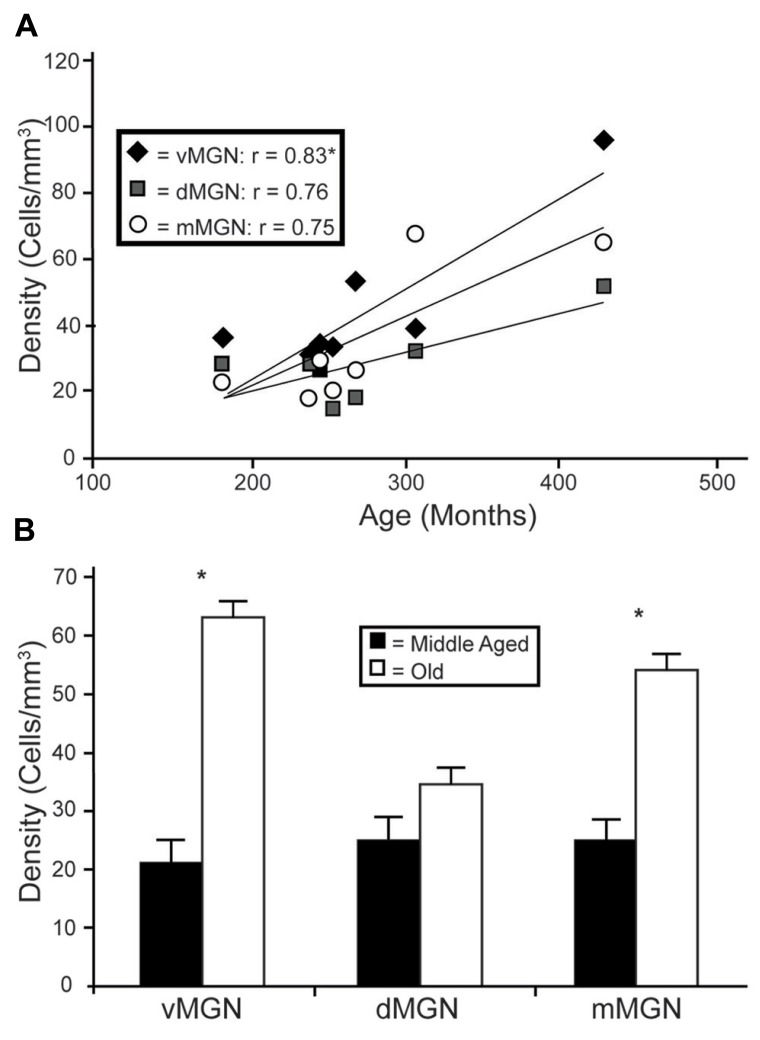
**Age-related changes in PV positive neuron density from the three subdivisions of the MGN.** **(A)** Regression analyses of the estimated cell densities from the ventral MGN (vMGN; black diamonds), dorsal MGN (dMGN; gray squares), and magnocellular MGN (mMGN; white circles) as a function of age showed a significant correlation only in the vMGN. **(B)** Estimated cell densities of the vMGN, dMGN, and mMGN when the animals were combined into middle aged and old age groups revealed significant increases in both the vMGN and mMGN. Asterisks indicate significance by an unpaired *t*-test at an alpha level of 0.01.

Visual inspection of the MGN revealed different patterns of age-related changes in PV expression in the three subdivisions. The density of PV+ cells was clearly increased in the vMGN and mMGN as a function of age, whereas changes in the dMGN were much less apparent (**Figure 2**). Quantifications of PV+ cell densities within these subdivisions revealed that our qualitative observations were accurate. A regression analysis of these densities as a function of age revealed that the number of PV+ neurons positively correlated with age (*r* = 0.83, *p* < 0.01 **Figure 3A**), whereas the PV densities of the mMGN and dMGN trended but did not significantly correlate with age by our criteria (*r* = 0.75, *p* = 0.027; *r* = 0.76, *p* = 0.021; mMGN and dMGN respectively; **Figure 3A**). Grouping the monkeys into middle aged and old age groups (see Methods) revealed that both the vMGN and dMGN significantly increased their densities of PV+ neurons (unpaired *t*-tests, *p* < 0.01; **Figure 3B**), but PV+ neuron density in the dMGN remained constant (unpaired *t*-test; *p* = 0.20; **Figure 3B**).

**FIGURE 4:**
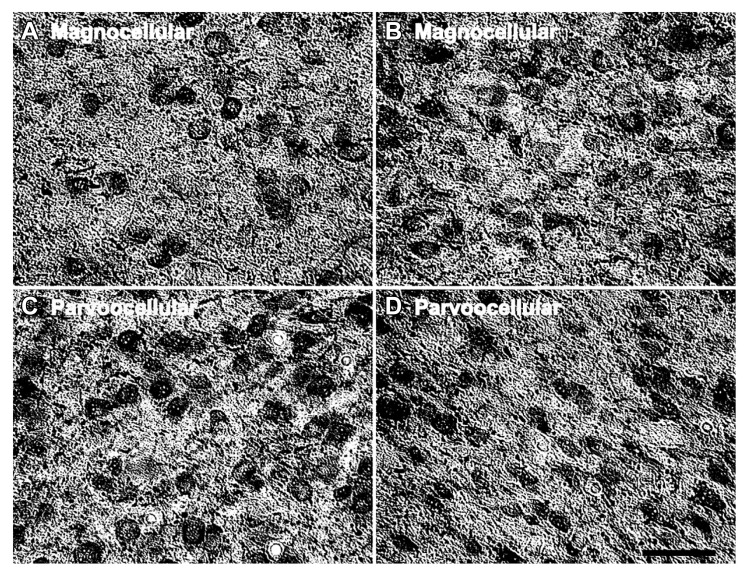
**Age-related changes in the number of parvalbumin positive cells in the LGN.** Comparison of micrographs from a **(A)** 15 year old magnocellular layer 2 and **(B)** 35 year old magnocellular layer 2 reveals apparent age-related increases in the number of PV+ cells. Likewise, a comparison of micrographs from a **(C)** 15 year old parvocellular layer 3 with a **(D)** 35 year old parvocellular layer 3 show similar increases. The left side of this image corresponds to the medial aspect and the top to the dorsal aspect of the sections. Scale bar = 150 μm.

**FIGURE 5:**
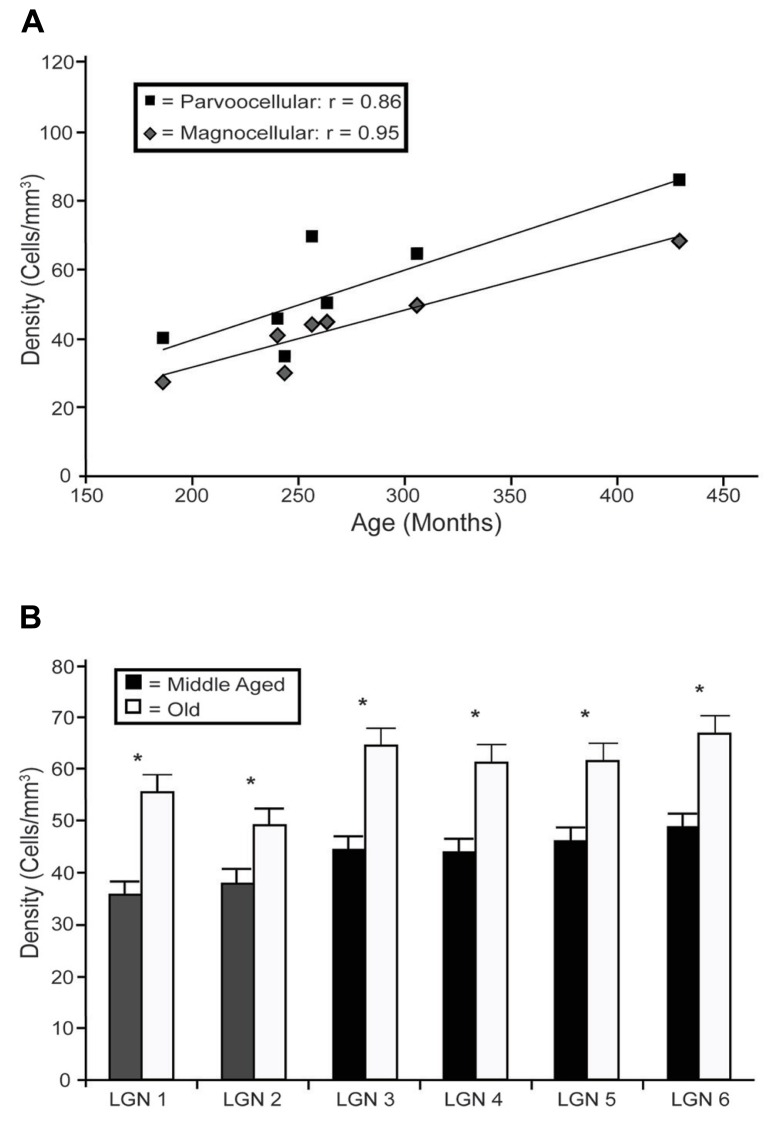
**Age-related changes in PV positive neuron density from the layers of the LGN.** **(A)** Regression analyses of the estimated densities of PV+ labeled cells from the magnocellular (layers 1 and 2; gray squares) and parvocellular (layers 3 through 6; black diamonds) layers as a function of age showed significant correlations in all layers with the exception of layer 5. **(B)** Estimated densities of the six individual layers when the animals were combined into middle aged and old age groups revealed significant increases in all LGN layers. Asterisks indicate significance by an unpaired *t*-test at an alpha level of 0.01.

In the LGN there appeared to be an increased density of PV+ neurons as a function of age in all six layers. **Figure 4** shows representative photomicrographs of magnocellular and parvocellular layers from a young and an old animal, and the age-related increases in the densities of PV+ neurons are apparent in both layers. Regression analysis (**Figure 5A**) of our stereological calculations revealed that both the magnocellular and parvocellular layers had increased density of PV+ neurons with age (*r* = 0.95, *p* < 0.01; *r* = 0.86, *p* < 0.01; magnocellular and parvocellular, respectively). Individually, each layer increased its expression with age (all *r* > 0.8; all *p* < 0.01; data not shown), except layer five which showed strong trends (*r* = 0.75; *p* = 0.051). Comparisons between the middle-aged and aged groups revealed an age-related increase in PV density across all layers (all *p* < 0.01; **Figure 5B**).

**FIGURE 6:**
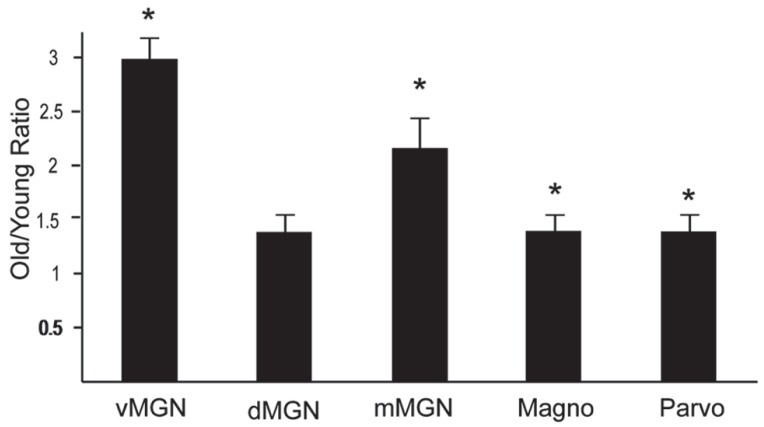
**Density of PV + neurons increases more in the MGN than the LGN.** Normalized PV density increases in the ventral, dorsal and magnocellular MGN (vMGN, dMGN, and mMGN; respectively), and from the magnocellular and parvocellular layers of the LGN. Values were found by taking the ratio of the old to young PV densities in each subdivision. Asterisks mark the regions that showed statistically significant age-related increases in the density of PV+ neurons. Asterisks indicate significance by an unpaired *t*-test at an alpha level of 0.01.

The preceding analysis showed that most MGN subdivisions and LGN layers from aged macaques have higher PV+ densities compared to those from middle aged macaques. However, since there was a higher density of PV+ neurons in the LGN compared to the MGN in the middle aged animals (compared **Figures 3B–5B**), it is not clear if the relative differences in densities as a function of age were the same between the different divisions of these two thalamic nuclei. In order to determine if this was the case, we normalized the differences in the aged monkeys by taking the ratio of the old PV densities to the middle aged PV densities. **Figure 6** shows the results of this analysis in the three subdivisions of the MGN and the magnocellular and parvocellular layers of the LGN. The increases in the vMGN and mMGN were much greater in magnitude than the changes in the magnocellular and parvocellular layers of the LGN. These results show that the age-related increases in PV density differ between the auditory and visual systems. Furthermore, these results suggest that the age-related effect is not due to general differences of the aging nervous system (differences in vascularization, permeability of the individual cells to the histochemical processes, or differences in immunoreactivity as a function of age), but rather the changes are specific for the different subdivisions of the MGN and LGN.

## DISCUSSION

### AGE-RELATED DIFFERENCES IN PV IMMUNOREACTIVITY IN THE CENTRAL AUDITORY SYSTEM

The primary objective of this study was to determine whether the macaque MGN increases its PV expression with age as seen in the SOC (Gray et al., 2013a), and IC (unpublished observations). Our data did show a clear increase in PV expression with age in the macaque MGN, consistent with data from rodents (Ouda et al., 2008). With the addition of the present data, all subcortical auditory structures of both macaques and rodents have shown age-related changes in the expression of calcium-related proteins (references cited above for macaques; for rodents see O’Neill et al., 1997; Zettel et al., 1997; Idrizbegovic et al., 2003, 2004, 2006; Ouda et al., 2008, 2012). The current thalamic data in the macaque is consistent with what has been observed in the macaque SOC (Gray et al., 2013a) and IC (unpublished observations) in that not all subdivisions showed age-related changes. In the SOC, only the medial superior olive showed statistically significant increases, whereas the lateral superior olive only showed a trend toward increases, and the medial nucleus of the trapezoid body showed no changes. In the IC, the central nucleus and the peripheral cortex region showed age-related changes in PV+ density. Interestingly, the CN showed no such changes in PV+ density with age, although the density of neurons that stained positively for NADPH-diaphorase (NADPHd) did increase with age, and the density of PV+ cells was correlated with changes in ABR thresholds (Gray et al., 2013b). These combined results indicate that while calcium-associated proteins are differentially expressed throughout the ascending auditory system in the macaque, the first relay nuclei showing age-related influences on PV expression are the SOC and IC. Thus, these age-related effects appear to be restricted to a specific sub-pathway of the ascending auditory system in macaques. Why these particular sub-divisions are effected in this way by age while others are not is currently unclear. Other calcium dependent proteins, such as the nitric oxide synthase molecule NADPHd have also been shown to increase with age in macaque and rodent subcortical auditory structures (Yan et al., 1996; Soares-Mota et al., 2001) including the macaque CN (Gray et al., 2013b). Therefore other calcium binding- and/or dependent-proteins may increase their expressions in ascending auditory structures of aged macaques as seen in rodents, and are likely in some way compensating for the decreased neural output of the cochlea (Engle et al., 2013).

### CHANGES IN THE MGN WERE MORE SPECIFIC THAN IN THE LGN

The second purpose of this study was to determine if these neurochemical changes of the aged auditory system were similar or different to those of the aged visual system. The thalamus is the ideal structure to make these direct comparisons as both nuclei can be visualized in the same anatomical section (**Figure 1**) and differences in histological processing are minimized. Similar to the MGN, all six layers of the LGN increased their expression of PV positive neurons with age. While the PV+ densities observed in the LGN were overall higher than those seen in the three sub-divisions of the MGN, the increased densities as a function of age were relatively much higher in the MGN compared to the LGN. This implies that at least a part of the noted changes are specific to the area and/or sensory system, and not a general change.

The principle subdivisions of the MGN and each of the six layers of the LGN have distinct functional properties. The ascending auditory system can be segregated into two major parallel processing pathways that originate at the level of the CN, and segregate anatomically, chemically, and functionally in higher auditory regions (Jones, 2003). At the level of the MGN, these pathways are almost entirely segregated, and much of our understanding of the function of these dual pathways arose from thalamic and cortical studies (Hu, 2003). The lemniscal pathway ascends mostly through the vMGN and primarily concerns itself with relaying the auditory signal, whereas the non-lemniscal pathway primarily ascends through the dMGN and is involved with higher order functions such as cuing, emotional responses, and learning processes (Merabet et al., 1998; LeDoux, 2000; Komura et al., 2001; Matsumoto et al., 2001). Interestingly the mMGN shows characteristics of both pathways, and is often considered an intermediate. The immunoreactivity of these processing streams to calcium binding proteins shows pronounced segregation, with the lemniscal pathway being PV rich and the non-lemniscal pathway being calbindin (CB) rich. Since PV was the only calcium buffer used in the study, it comes as no surprise that only the vMGN and mMGN increased their expressions of this protein with age. Whether the dMGN increases expression of its characteristic calcium binding protein, CB, with age remains to be seen.

The layers of the LGN are also specific in their functional response properties, but not chemically specific to calcium binding proteins like the MGN. Magnocellular layers 1 and 2 and parvocellular layers 3 through 6 receive distinct input from the retina, and have differing response properties (for review see Gegenfurtner and Kiper, 2003). Despite these differences, all layers experienced similar age-related increases in PV immunoreactivity with age. Therefore the visual system showed less specificity than did the auditory system with regards to age-related increases in PV. Whether this pattern holds true elsewhere in the visual system as it does in the auditory system cannot be inferred from the present data. Also, it is plausible that another calcium binding protein would yield different patterns.

### SENSORY DEPRIVATION MODELS NATURAL AGING BETTER THAN DEAFFERENTATION

To our knowledge this is the first study investigating the effects of natural aging on PV immunoreactivity in the macaque visual system, and the results show similar increases in PV expression as seen in the auditory system. Deafferentation studies in both systems yield opposite results from the data reported here. Following monocular or binocular enucleations, the visual system decreases its expression of PV+ neurons both thalamically and cortically (Blümcke et al., 1994), and ablation of the cochlea results in decreases in the expression of calcium binding proteins as well (Hatano et al., 2009). Interestingly, sensory deprivation studies in both systems result in either unaltered or increased expression of calcium binding proteins (Mize and Luo, 1992; Tigges and Tigges, 1993; Caicedo et al., 1997), suggesting that deafferentation and sensory deprivation affect these systems differently and in opposite directions. Our data suggests that the effect that natural aging has on the immunoreactivity of calcium binding proteins resembles the effects of sensory deprivation rather than deafferentation. This distinction is important because both sensory deprivation and deafferentation are often implemented to mimic the natural declines in peripheral processing abilities associated with age. However, their effects on the nervous system are not analogous to the effects of natural aging, and must be considered accordingly.

## Conflict of Interest Statement

The authors declare that the research was conducted in the absence of any commercial or financial relationships that could be construed as a potential conflict of interest.

## AUTHOR CONTRIBUTIONS

All authors had full access to all the data in the study and take responsibility for the integrity of the data and the accuracy of the data analysis. Study concept and design: Daniel T. Gray, James R. Engle, Gregg H. Recanzone. Acquisition of data: Daniel T. Gray, Megan L. Rudolph, James R. Engle. Analysis and interpretation of data: Daniel T. Gray, Megan L. Rudolph, Gregg H. Recanzone. Drafting of the manuscript: Daniel T. Gray, Gregg H. Recanzone. Critical revision of the manuscript for important intellectual content: Daniel T. Gray, Megan L. Rudolph, Gregg H. Recanzone. Statistical analysis: Daniel T. Gray, Megan L. Rudolph. Obtained funding: James R. Engle, Gregg H. Recanzone. Administrative, technical, and material support: Daniel T. Gray, James R. Engle. Study supervision: Gregg H. Recanzone.
